# What do Australian consumers, pharmacists and prescribers think about documenting indications on prescriptions and dispensed medicines labels?: A qualitative study

**DOI:** 10.1186/s12913-017-2704-3

**Published:** 2017-11-15

**Authors:** Mona Garada, Andrew J. McLachlan, Gordon D. Schiff, Elin C. Lehnbom

**Affiliations:** 1 Faculty of Pharmacy, University of Sydney, Sydney, Australia; 2 Centre for Education and Research on Ageing, Concord Hospital, Sydney, Australia; 3 Division of General Internal Medicine and Primary Care, Department of Medicine, Brigham and Women’s Hospital, Boston, MA USA; 4 Medical Management Centre, Department of Learning, Informatics, Management and Ethics, Karolinska Institutet, Stockholm, Sweden; 5 Department of Pharmacy, Faculty of Health Sciences, UiT – The Arctic University of Norway, Tromsø, Norway

**Keywords:** Drug labelling, Prescription drug, Qualitative study, Medication indication

## Abstract

**Background:**

Documenting the indication on prescriptions and dispensed medicines labels is not standard practice in Australia. However, previous studies that have focused on the content and design of dispensed medicines labels, have suggested including the indication as a safety measure. The aim of this study was to investigate the perspectives of Australian consumers, pharmacists and prescribers on documenting the indication on prescriptions and dispensed medicines labels.

**Methods:**

Semi-structured interviews were conducted and mock-up of dispensed medicines labels were designed for participants. Consumers (*n* = 19) and pharmacists (*n* = 7) were recruited by convenience sample at community pharmacies in Sydney (Australia) and prescribers (*n* = 8), including two medical students, were recruited through snowballing.

**Results:**

Thirty-four participants were interviewed. Most participants agreed that documenting the indication would be beneficial especially for patients who are forgetful or take multiple medications. Participants also believed it would improve consumers’ medication understanding and adherence. Prescribers and pharmacists believed it could help reduce prescribing and dispensing errors by matching the drug/dosage to the correct indication. Prescribers refrained from documenting the indication to protect patients’ privacy; however, most patients did not consider documenting the indication as a breach of privacy. Prescribers raised concerns about the extra time to include indications on prescriptions and best language to document indications, using plain language as opposed to medical terminology.

**Conclusions:**

All interviewed stakeholders identified numerous benefits of documenting the indication on prescriptions and dispensed medicines labels. Whether these potential benefits can be realized remains unknown and addressing prescribers’ concern regarding the time involved in documenting the indication on prescriptions remains a challenge for vendors of electronic medication management systems.

**Electronic supplementary material:**

The online version of this article (10.1186/s12913-017-2704-3) contains supplementary material, which is available to authorized users.

## Background

The guidelines for labelling dispensed medicines in Australia requires information such as the directions for use, the dose form and the quantity supplied to be clearly visible on the label. [1] However, neither the prescription nor the dispensed medicine label requires the documentation of the indication or *purpose* of the medication. The Australian National Inpatient Medication Chart (NIMC) is a paper and electronic chart that health-care professionals use in public hospitals to document information (e.g. route, dose) on the medications of an inpatient [2]. The NIMC includes a section where the indication of a medication can be documented, but compliance is low (8.9%) [3]. The dispensed medicine label, along with a Consumer Medicine Information (CMI) leaflet, is often the final mode of communication between the prescriber, pharmacist and consumer. Ensuring the dispensed medicine label contains adequate information for the consumer to refer to for the medicine’s appropriate use is thus crucial. Unfortunately, consumers and pharmacists may be unsure about the specific indication for a dispensed medication [4].

Including the indication is common practice in some European countries [5]. In Sweden for example, the indication must be written in lay Swedish terms on a dispensed medicine label so consumers can understand what the medication is to be used for [5]. A retrospective study of prescription errors in Swedish community pharmacies found that having the indication documented on the prescription helped pharmacists identify and rectify prescription errors [6].

Previous qualitative studies, which have focused on the content and design of the dispensed medicine label, have found that 28% to 91% of patients were in favour of documenting the indication on the dispensed medicine label [7–13]. Additionally, two literature reviews on best label practice, comprising the results of 135 individual studies, found that consumers *want* the indication documented on the dispensed medicines labels to better understand/remember what the medications are used for [14, 15]. Furthermore, a prospective descriptive study conducted in Australia over 20 years ago explored the views of prescribers, pharmacists and consumers on new prescription layouts [16]. The proposed prescription layout at the time included a section in which the general practitioner (GP) could document the indication of the medication [16]. In the study, 35% (*n* = 1222) of the new prescription layouts had the indication documented as part of the written directions [16]. Even though it has been known for many years that consumers, pharmacists and GPs feel that documenting the indication on the prescription and the dispensed medicine label could lead to better medication management and patient care [16], this is still not common practice in Australia.

The aim of this study was to investigate current perspectives of various Australian stakeholders, including consumers, pharmacists and prescribers, regarding documenting the indication on prescriptions and dispensed medicines labels.

## Methods

This qualitative study was approved by the University of Sydney’s Human Research Ethics Committee (ref: 2016/443). Three slightly different semi-structured interview guides were developed for interviews with consumers, pharmacists and prescribers respectively (Additional file 1). The questions were constructed to gain insight into medication use (taking/not taking medications) and explore participants’ views on documenting the indication on the dispensed medicine label, how that information should be worded, and its potential effect on medication errors and patient safety. The interviewer (MG) conducted practice interviews with pharmacy students and members of the research team, before data collection begun. A number of mock-up dispensed medicines labels with different wording were created and presented to participants for consideration during the interviews (Additional file 2). This was done to ensure all participants understood by documenting the indication on dispensing labels, and to explore the preferred wording.

### Study participants and data collection

A convenience sample of consumers and pharmacists were recruited in community pharmacies whereas prescribers (general practitioners and hospital doctors) were recruited using snowball sampling from general practice and the public hospital setting. Eligible participants (taking, picking up, prescribing or dispensing medications and proficient in English) were informed verbally by the interviewer and provided a written information sheet outlining the purpose of study (to explore Australians’ views regarding documenting the indication on prescriptions and dispensing labels). Participants were interviewed once by one female interviewer (MG) as soon as written consent had been obtained. The interviews were conducted in quiet settings of the interviewees’ workplaces and no other person was present but the interviewer and interviewee. Interviews with consumers took place in a quiet area of the pharmacy. Only the interviewer and interviewee were present during the interview. Interviews were audio-recorded and transcribed *verbatim*. No field notes were taken when the interviews were audio-recorded. If the participant did not consent to audio-recording, the participants’ answers were handwritten during the interview. The audio-recorded interviews took an average of seven minutes and 43 s. Data collection took place in August – September 2015. Only one consumer who was approached declined to participate.

Participants were asked to complete a brief demographic questionnaire before the interview. The participant demographic form included information on the participants’ gender; age; number of prescription medications; whether they were a consumer, prescriber or a pharmacist and if the latter, their setting of practice (e.g. hospital, community, public sector, private sector).

### Data analysis

Transcribed interviews were imported to NVivo 11 (NVivo qualitative data analysis software; QRS International Pty, V11). Initial data coding was undertaken in parallel with data collection. Data collection ceased when no new themes were being identified, indicating that data saturation had been achieved [17].

Applying the framework method [18], the data were coded using NVivo. Codes with similar meaning were grouped together into different categories, and a total of four themes were identified on documenting the indication on prescriptions and dispensed medicines labels; the potential benefits, the potential limitations, describing the indication on the dispensed medicine label and the safety improvements.

## Results

A total of 34 participants were interviewed and their demographic details are shown in Table 1. All but two interviews were audio-recorded. At least one medication was taken by 65% of the participants. A summary of key points for each theme is shown in Table 2.

**Table 1:** Participant demographics

		Number of participants (%)
Gender	Female	27 (79.4)
Age (years)	≤30	9 (26.5)
	31–40	5 (14.7)
	41–50	7 (20.6)
	51–60	5 (14.7)
	>60	8 (23.5)
Number of prescription medications	None	12 (35.3)
	1–5	22 (64.7)
Occupation	Consumer	19 (55.9)
	Pharmacist	7 (20.6)
	Prescriber	6 (17.6)
	Medical student	2 (5.9)
Occupation setting (for HCPs)	Hospital	7 (46.7)
	Community	6 (40.0)
	Private sector	2 (13.3)

**Table 2:** Key theme points identified

Theme	Key Points
Potential benefits	• Reminder to take medications • Help when picking up medications on behalf of someone else • Useful with non-tablet medications (e.g. creams) • May encourage health checks • Medication reconciliation • Helps with the management of a patient in emergency situations • Helps when medication has multiple indications
Potential limitations	• Privacy concerns • Overcrowding of the label • Prescriber difficulty with defining/clarifying indication
Describing the Indication	• Medical terminology may make consumer take condition more seriously • Treatment specificity preferred with anti-infectives
Potential Safety Benefits	• May reduce confusion with generic brand substitutions • Helps health-care professional match dose to indication • May reduce look-alike sound-alike errors

### Potential benefits of documenting the indication on dispensed medicines labels

Most of the consumers reported that documenting the indication on the dispensed medicine label would help them identify what their medications were for. These participants perceived having the indication on dispensed medicines labels would make managing medication less confusing, especially for those on multiple medications and for people who are forgetful. The idea of documenting the indication on the dispensed medicines label was perceived as particularly important when starting a new medication or when an alternative brand was dispensed as consumers reported being more confused in these situations. One consumer (#15) reported: “*…you have more information, it can only help*”. Another consumer (#14) agreed and added: “*I look at it as just another positive [piece of] information they’re getting…*”.

Most consumers not only perceived having the indication on the dispensed medicines label as beneficial for themselves, but also for their partners and other caregivers. For example, individuals who collect medications on behalf of someone else thought it would be useful for everyone involved if the indication was documented on the dispensed medicine label. Consumers also felt it was beneficial to document the indication on the dispensed medicine label for topically applied medicines (e.g. creams) as well as eye and ear drops because these products can be used for different health conditions by different consumers.

However, not all consumers felt it was necessary to document the indication on the dispensed medicine label. In fact, some consumers felt that if healthcare professionals required the indication to be documented, they would be worried. As one consumer (#19) stated, “*I think if my doctor needed to have that I would be worried, because they should know what things are for!*” Some consumers were indifferent to the notion of documenting the indication, but most were in agreement that it would be “good”.

Most of the pharmacists in the study stated that documenting the indication on the dispensed medicine label would be beneficial as consumers would be “*clued in to what conditions they have*,” (Pharmacist #6), “*it helps patients just taking control over their own medications, knowing what they’re taking and why, having that information is power to them*,” (Pharmacist #7) and “*the consumer themselves are more hungry for information these days*” (Pharmacist #7). Some pharmacists stated that it would be helpful to document the indication on the dispensed medicine label for older patients who are prescribed many different medications. As one pharmacist (#5) stated: “*…possibly for those people that we assess who don’t fully understand or maybe those patients who are a bit…and I’ll generalize…a little bit older…to help for their understanding of their therapy treatment…*” Additionally, pharmacists reported it would be beneficial to document the indication on the prescription when the medication has multiple indications or when the medication is being used “off-label”.

Most of the pharmacists also identified having the indication on dispensed medicines labels as something positive when consumers had old repeat (refill) prescriptions for an antibiotic and were deciding to fill it in when they were not feeling well. *“…often they would bring a script from, you know, six months ago, or something, for an antibiotic, which they can’t remember what it was prescribed for…and then they’ll say, ‘I’ve got this terrible cough, will this work for me?’, I think it would help to have an indication.*” (Pharmacist #3) However, other pharmacists viewed counselling as more important and believed it sufficient to educate the patient on the indications of their medications.

Prescribers reported that documenting the indication would be worthwhile as it would assist in sustaining continuity of care for patients who were being treated by multiple healthcare professionals. Prescribers practicing in hospitals reported that if the indication was documented on the dispensed medicine label, it would help with medication reconciliation. Currently, prescribers make “*educated guesses*” (prescriber #1) or ask the patient to explain what their medications are for. Additionally, one prescriber (prescriber #1) reported that knowing the indication, for example whether metoprolol was being used for heart failure or hypertension, would impact on the management of patients.

#### Possible impact on medication adherence

Most of the participants reported that documenting the indication would make them “*more inclined to*” (consumer #14) take their medications thus improve their medication adherence. Healthcare professionals, especially prescribers, tended to agree. A prescriber said that conditions such as *“…blood pressure or diabetes, it’s more based on…blood test results or blood test readings that they need to be on the tablets, it’s not how they* feel*…*,” (prescriber #6); and so, documenting the indication would help remind them of their condition and encourage them to continue to take the medication as prescribed. One medical student stated that perhaps by documenting the indication of conditions that are asymptomatic and require tests, such as hypertension, would encourage consumers to have regular check-ups. In addition, one consumer (consumer #11) believed that documenting the indication for a cholesterol-lowering medication can encourage individuals to maintain their cholesterol levels “*within acceptable limits*”.

### Potential limitations of documenting the indication on dispensed medicines labels

When asked what could be negative about documenting the indication on the dispensed medicine label, patients felt that the dispensed medicine label is already overcrowded and contains too much information. One consumer believed it would be good to document the indication “…*as long as it doesn’t get lost in all the small print, because a lot of people aren’t going to read it anyway*,” (consumer #11). Not reading the label, hence missing important information, was another potential limitation identified by consumers. As one consumer stated, “*Yeah, but when you got ten or a dozen of these, you’re not continually reading all labels*…,” (consumer #16). Consumers also reported that the small font on the dispensed medicine label would discourage them from referring to it.

Prescribers expressed concerns about the time it would take to document the indication on the prescription and how challenging it would be to find that time in an already time pressure environment. Interestingly, prescribers stated that documenting the indication for medications that had, what they perceived to be, an *obvious* indication, such as a statin for hypercholesterolemia, would be a nuisance and unnecessary. Prescribers, including the medical students, thought it would also be a challenge to *clarify*, or rather narrow down, the exact indication for a medication that can be used for multiple indications.

#### Privacy concerns

Privacy was mentioned multiple times by participants when talking about potential limitations of documenting the indication on dispensed medicine labels. One prescriber referred to this practice as a possible “*breach of confidentiality*” (prescriber #2). Specifically, conditions such as mental health conditions, sexual dysfunction, sexually transmitted diseases (e.g. genital herpes), cancer and HIV were mentioned as particularly problematic. Healthcare professionals acknowledged this and suggested strategies whereby patients had to provide consent to having the indication documented on prescriptions and dispensed medicines labels.

Interestingly, most of the consumers said they would be comfortable with having the indication documented on the dispensed medicine labels even for sensitive conditions because healthcare professionals already *know* what medical conditions they have, hence it would not be a privacy issue. However, some consumers did not want their family members to know the exact indication for some of the medications they were taking hence did not want the indication documented. On the other hand, other consumers stated that privacy “*goes out the door*” when you are living with someone, and thus privacy would not be an issue when it comes to documenting the indication. Some participants also reported that disposing the medication box with the indication on the label could potentially result in a breach of privacy if an unauthorised person, such as a neighbour, found the medication box.

### Describing the indication on dispensed medicines labels

Almost all participants preferred the mock-up dispensed medicine label written in plain English without any medical terms or jargon. For example, most patients preferred “…to lower blood pressure,” as opposed to “…for hypertension.” Healthcare professionals thought it would be best to keep it as simple as possible for the consumer. However, some prescribers believed that by documenting the indication in medical terms, such as “psoriasis” as opposed to a “skin rash” or “for severe COPD” instead of “difficulty breathing” would make the patient take their condition more seriously and comply better to the medication.

When participants were asked about how specific the indication should be (e.g. infection or urinary tract infection), most participants stated that it would be better to be more specific. However, some consumers stated that for a short-term course of an anti-infective, documenting the indication would not be necessary as they would know what infection they had, hence what the medication was being used for. Pharmacists on the other hand believed documenting the specific indication for anti-infectives would lead to more appropriate use of antibiotics. That is, if the label clearly stated that the medication was for “urinary tract infection” rather than “infection” consumers might be discouraged from inappropriate self-medicating.

### Potential safety benefits

Most participants felt that documenting the indication has a role in preventing medication errors and reducing or avoiding harm. Participants felt that the indication could be somewhat of a safety check for healthcare professionals and consumers alike. Pharmacists believed that it may reduce prescribing and dispensing errors of ‘look-alike, sound-alike’ medications. One pharmacist (#4) gave the example of allopurinol sounding very similar to haloperidol, which are used for completely different conditions. Prescribers reported that errors could be reduced by matching the correct drug and dosage for the correct indication. A medical student (#2) stated that: *“…they* [the pharmacists] *would have an idea of the dosages for different conditions, like if a patient is on doxycycline for an acute infection it might be different to doxycycline for malaria or for…acne…*”. Another prescriber (#6) stated: “*I have seen that in the past, where they have been given the wrong dose because that dose is for a different condition…so if it came up what the indication was, there’s also double checking the right dose, yeah. I can see that helping*”.

## Discussion

This study investigated the perspectives of consumers, pharmacists and prescribers, on documenting the indication on the prescription and dispensed medicines labels. In this study, most participants expressed positive views about documenting the indication on prescriptions and dispensed medicines labels.

Despite the potential benefits participants identified with including the indication, privacy was a concern raised by all. Similar concerns were raised by participants in a Dutch study, where consumers did not want conditions such as sexually transmitted infections, cancer or even onychomycosis to be documented on the dispensed medicine label [8]. Interestingly, even though consumers discussed privacy concerns in general, Australian consumers appeared accepting of documenting the indication for a sensitive or stigmatized condition if the medication was for *themselves.* In our study, it was mainly prescribers and pharmacists who worried about consumers’ privacy indicating that healthcare professionals’ concern may outweigh consumers’ actual concern and fears, and that consumers’ privacy can be maintained by simply asking them whether or not they want the indication documented on prescriptions and dispensed medicine labels.

Some pharmacists believed that verbal medication counselling is more important than documenting the indication on the dispensed medicine label. We are not suggesting that documenting the indication on dispensed medicine labels would replace medication counselling, but can complement verbal counselling thereby providing an added safety measure to improve consumer medication understanding and reduce prescribing and dispensing errors. In fact, a study in which the indication was documented on the prescription for pharmacists helped pharmacists confirm the medication was correct for the indication provided [19]. Knowing the indication may also facilitate appropriate medication counselling whereby the pharmacist can tailor the information and make it more “patient-centred”. Previous studies have reported that pharmacists preferred to have the indication on the prescription to avoid having to ask the consumer or guess the indication [11, 20]. Moreover, in the study by Liddell and Goldman [16], prescribers and pharmacists believed that documenting the indication would improve the quality of information and minimize irrelevant and inappropriate advice as well as provide consistent advice from all healthcare professionals. Pharmacists also reported in a previous study that being provided the indication on prescriptions helped reduce consultation time with the prescriber in order to clarify or correct prescriptions from 38.7% (*n* = 22/57) to 61.5% (*n* = 8/13); hence decreasing interruptions for prescribers [19]. Previous research have found that pharmacists make better clinical decisions if they know the diagnosis or reason for prescribing [21]. This holds true for electronic prescriptions as well [19].

Warholak-Juarez et al. studied how additional patient information given to a pharmacist would affect their clinical decision making [21] and found that pharmacists were able to make better clinical decisions when they had more patient information available to them, such as the diagnosis/problem (i.e. purpose of the medication) [21]. The less information pharmacists had about the patient, the more they realized the assumptions they were making [21]. Compared to having complete patient information to incomplete information, pharmacists in the study by Warholak-Juarez et al. were able to realize the risk they could have put a patient in when making clinical decisions without complete patient information [21]. This further supports the positive effect of having the indication documented on a prescription.

The wording of the indication on the prescription and dispensed medicine label was a source of concern among the participants in this study. Previous studies have shown that consumers prefer written information to be in plain language to help their medication understanding and management [12, 22, 23]. A previous review also had healthcare professionals agreeing to keep written information in plain language [23]. Similarly in our study consumers favoured simple, plain language; however some healthcare professionals wanted to use more medical terms. However there is an issue of how the indication can best be documented—should it be by symptom, health problem, diagnosis, ICD-10 or SNOMED-CT [4]. A study trial conducted on indication-based prescribing of antihypertensive medications allowed prescribers use either free text or common discrete codes of ICD-9 CM or SNOMED [24]. The study highlighted that the technology to process free text clinical documentation is promising but requires optimisation [24]. Agreeing on the terminology to be used will enable vendors to incorporate shortlists of indications and other technical support in software that will facilitate safe and efficient documentation of indication when prescribing and dispensing medications.

Another issue that would create problems for prescribers including the indication on prescriptions is that they do not always know what indication they are treating. Schiff and colleagues note that there is a “complexity in defining and creating indications” for prescribing systems as well as the use of empirical treatments with medications when there is no definite diagnosis [4]. However this need not necessarily mitigate against the need and value of indications-based prescribing, only for developing approaches that help overcome these issues. A previous study on indication-based prescribing systems has shown that an indication prompts may be able to improve problem list documentation [24], and by doing so, perhaps an indication-based prescribing system could assist prescribers to prescribe more appropriately in these situations. Another study on indication-based prescribing for off-label medications was encouraging in showing that decision support software could be deployed to help recommend on-label alternatives or off-label alternatives with a higher degree of evidence when the indication is entered by clinicians [25].

One promising approach to documenting the indication on prescriptions is to design and implement an indication-based prescribing system in which the prescriber first select an indication (rather than the drug) and the prescribing system would help narrow down the choices of medications to those indicated for that condition [4]. A smart system would even be able to incorporate other patient specific information such as medications previously tried and failed or renal status that might contraindicate (and thus not include on the list of choices) a medication. A previous study on indication-based prescribing systems has shown that indication prompts can potentially intercept wrong charting of medications and intercept drug name confusion errors [24].

In summary, the findings from this qualitative study suggest that key stakeholders including consumers, pharmacists and prescribers in Australia all support the notion of including the indication on prescriptions and dispensed medicine labels. Future studies should investigate the impact of documenting the indication on medication error rates and patient outcomes. These may include a randomized control trial to measure the effects of documenting the indication on patient medication knowledge, adherence rates and clinical outcomes; a study using a mystery shopper design to assess pharmacists’ ability to detect prescribing errors on prescriptions with and without the indication; and an evaluation of different prescribing and dispensing software.

The strengths and weaknesses of this study include a number of factors. This study comprised 34 participants, providing ample qualitative data to explore the topic. Another strength was the inclusion of different stakeholders. However, most participants were from metropolitan New South Wales regions of Australia, limiting the generalisability to other settings. However our findings closely mirrored those of other studies and commentaries on this subject.

## Conclusions

This study identified and reinforced a number of potential benefits to documenting the indication on the prescription and the dispensed medicine label which participants felt outweigh any potential limitations. The main perceived benefits of including the indication were improved medication management, better understanding of medications among consumers, improved medication adherence and a possible reduction in medication errors. Changing prescribing practices represents a fundamental change hence the potential workflow and practice barriers, particularly time to document and privacy concerns, need to be addressed to fully realize the potential of incorporating indications into prescriptions and dispensed medication labels, although there are likely creative ways these barriers can be effectively overcome.

## Additional files

Additional file 1: Semi-structured interview guides. (DOCX 17 kb)

Additional file 2: Sample dispensed medicines labels with addition of indication. (DOCX 642 kb)

## Abbreviations

CMI: Consumer Medicine Information
COPD: Chronic obstructive pulmonary disease
GP: General practitioner
HIV: Human immunodeficiency virus
ICD-10: International Classification of Diseases, tenth revision
NIMC: National Inpatient Medication Chart
SNOMED-CT: Systematized Nomenclature of Medicine – Clinical Terms
