# Supplementary material for: What do Australian consumers, pharmacists and prescribers think about documenting indications on prescriptions and dispensed medicines labels?: A qualitative study
Source: BMC Health Serv Res. 2017 Nov 15;17:734. doi: 10.1186/s12913-017-2704-3 (PMC5688705; doi:10.1186/s12913-017-2704-3)
Supplement: Supplementary file 1 — Semi-structured interview guides. (DOCX 17 kb) [file 12913_2017_2704_MOESM1_ESM.docx]

**Additional file 1**

Semi-structured interview guide - **consumers**

1. Can you tell me about your experiences with prescription medications?
   1. Are you currently taking any regular medications?
   2. Do you know what they are for?
2. How would you feel if the doctor wrote the indication on your prescription so the pharmacist could write that on the label? For example, take one tablet at night for high cholesterol.
   1. Can you see any benefits of having the indication on prescriptions? Prompts: reduced risk of prescribing and dispensing errors, improved consumer knowledge.
   2. Are there any challenges? Prompts: privacy, legal, off-label prescribing, time.
3. If the indication is documented, how would you prefer it to be documented? For example, take this medication for hypertension or for blood pressure.

In some countries doctors have to write the indication on prescriptions. This is to prevent medication errors and help patients remember what their different medications are for.

[If asked: Some medications have names that look and sound alike but are used for different conditions. For example, if written quickly, anafranil (an antidepressant) and enalapril (for high blood pressure) may look alike leading to a dispensing error.]

1. Would documenting the indication of the medication help you remember take your medications regularly?
2. Would it make a difference if you had multiple medications for the same purpose? For example, three different medications are given for blood pressure?
3. How would documenting the indication on sensitive conditions affect your opinion on this? For example, conditions such as cancer or HIV?
4. How many medications are you on? If I asked you right now would you be able to tell me what each of them are for?
5. Do you think the length of treatment of the medication would affect your preference for documenting the indication? For example, documenting the indication for a short-term infection such as a urinary tract infection or documenting the indication for a chronic condition such as diabetes.
6. Can you think on any patient groups that may find this helpful/embarrassing?

[Patients on multiple medications, elderly patients, people with poor memory, people with stigmatised conditions (HIV, hepatitis, schizophrenia), or “embarrassing” conditions (erectile dysfunction)].

1. Anything else you’d like to tell me?

Semi-structured interview guide – **prescribers**

1. How often would you say you include the indication on prescriptions?
2. Why do/don’t you include the indication?
3. Do you write it in medical (hypertension) or lay (for high blood pressure) terms?
4. What are the benefits/drawbacks of having the indication on prescriptions? Prompts: integrating problem list with medication history, reducing calls to and from pharmacy, decreasing sound alike/look alike prescribing errors, aiding care transitions.
5. How important is it for consumers to know the indication of the medications they are taking?
6. Do you think having the indication on prescription aids medication adherence and patient understanding?
7. How important is it to move this forward?
8. Would documenting the indication be overwhelming to the patient?
9. What needs to happen in order for this to become standard practice?
10. How important do you think it is? And how likely?
11. Anything else you’d like to tell me?

Semi-structured interview guide – **pharmacists**

1. How often do you see a prescription with the indication written on it?
2. How often do/don’t you include the indication on the label?
3. Do you write it in medical (hypertension) or lay (for high blood pressure) terms?
4. What are the benefits/drawbacks of having the indication on prescriptions? Prompts: integrating problem list with medication history, reducing calls to and from pharmacy, decreasing sound alike/look alike prescribing errors, aiding care transitions.
5. How important is it for consumers to know the indication of the medications they are taking?
6. Do you think having the indication on prescription aids medication adherence and patient understanding?
7. How important is it to move this forward?
8. What needs to happen in order for this to become standard practice?
9. How important do you think it is? And how likely?
10. Do you often need to ask the patient why the doctor has given this certain medication?
11. If you were to document the indication, would you guess the reason it was given or would you ask the patient beforehand?
12. Anything else you’d like to tell me?
