# Supplementary figures and images for: What do Australian consumers, pharmacists and prescribers think about documenting indications on prescriptions and dispensed medicines labels?: A qualitative study
Source: BMC Health Serv Res. 2017 Nov 15;17:734. doi: 10.1186/s12913-017-2704-3 (PMC5688705; doi:10.1186/s12913-017-2704-3)

**Additional file 2**

Sample dispensed medicines labels with addition of indication


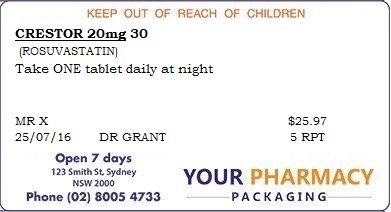

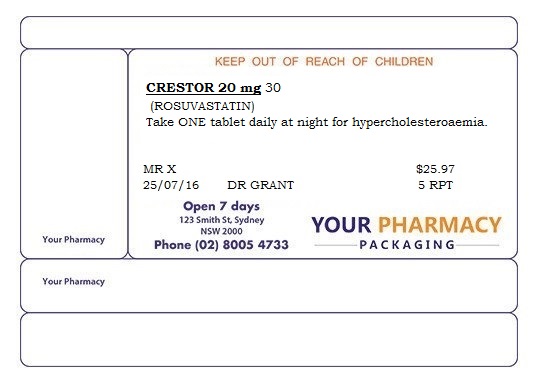


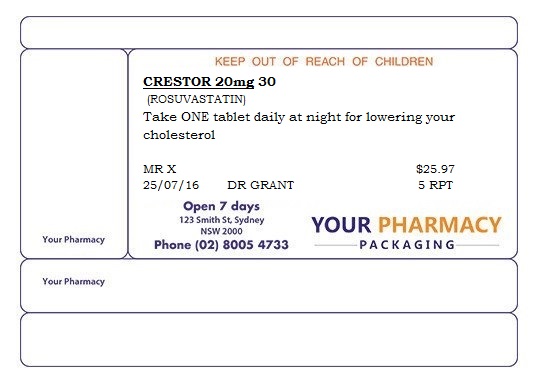


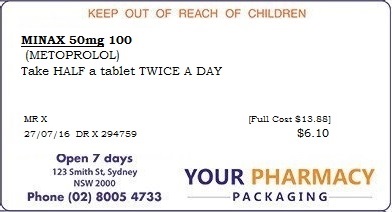

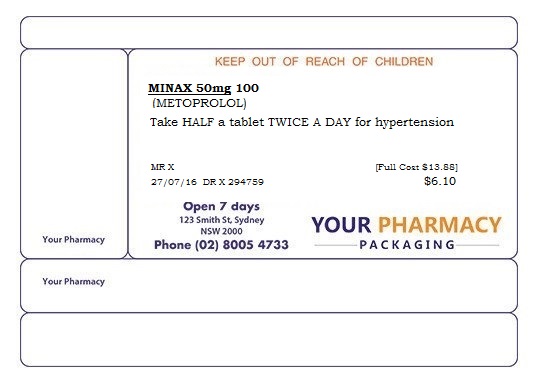


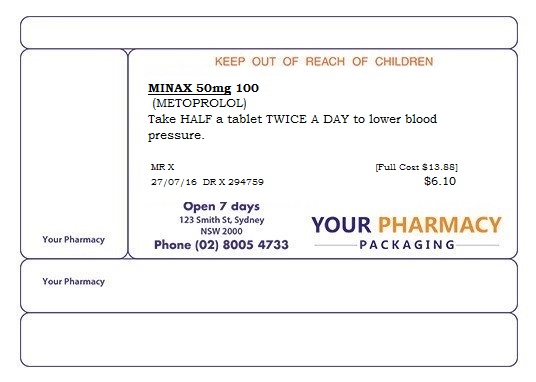


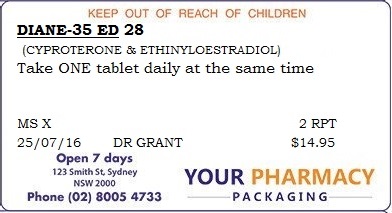

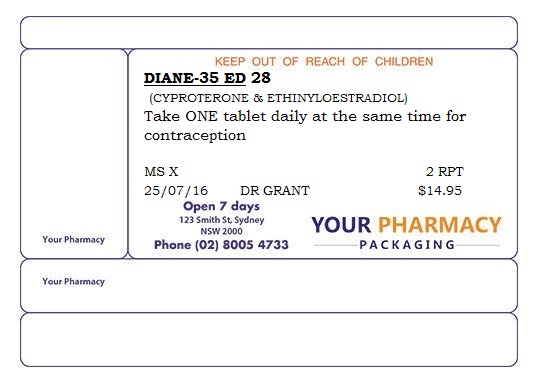


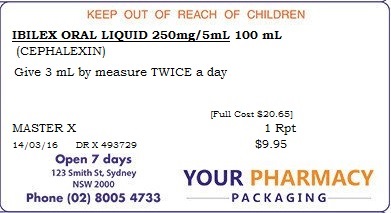

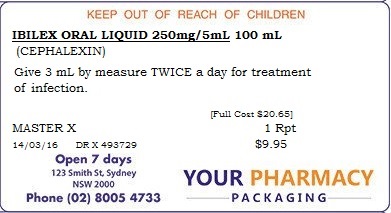


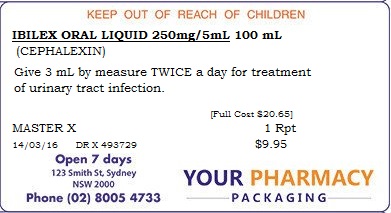


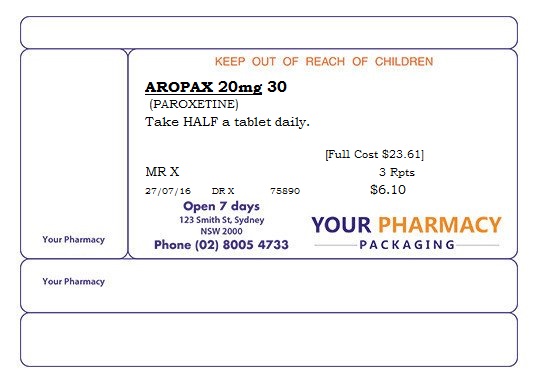

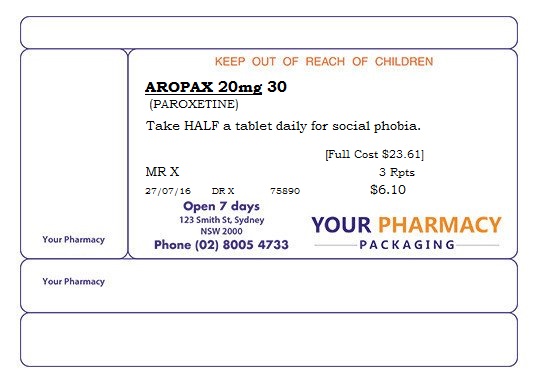


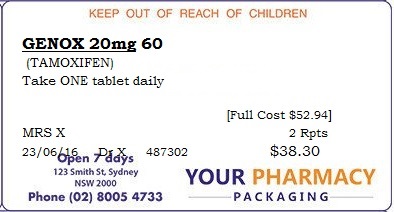

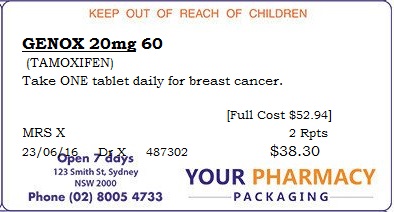


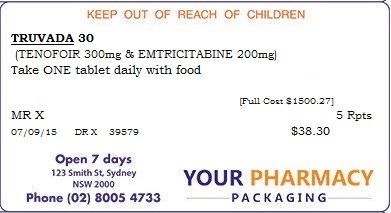

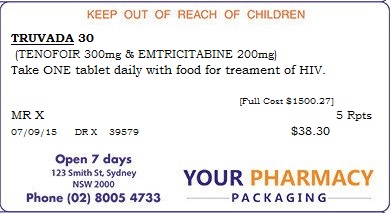

Supplement: Supplementary file 2 — Sample dispensed medicines labels with addition of indication. (DOCX 642 kb) [file 12913_2017_2704_MOESM2_ESM.docx]
